# Photochromic and Photocatalytic Properties of Ultra-Small PVP-Stabilized WO_3_ Nanoparticles

**DOI:** 10.3390/molecules25010154

**Published:** 2019-12-30

**Authors:** Daniil A. Kozlov, Alexander B. Shcherbakov, Taisiya O. Kozlova, Borislav Angelov, Gennady P. Kopitsa, Alexey V. Garshev, Alexander E. Baranchikov, Olga S. Ivanova, Vladimir K. Ivanov

**Affiliations:** 1 Lomonosov Moscow State University, Leninskiye Hills 1, 119234 Moscow, Russia; kozlov@inorg.chem.msu.ru (D.A.K.); taisia.shekunova@yandex.ru (T.O.K.); garshev@inorg.chem.msu.ru (A.V.G.); a.baranchikov@yandex.ru (A.E.B.); 2 Kurnakov Institute of General and Inorganic Chemistry of the Russian Academy of Sciences, 31 Leninsky av., 119991 Moscow, Russia; runetta05@mail.ru; 3 Zabolotny Institute of Microbiology and Virology, National Academy of Sciences of Ukraine, D0368 Kyiv, Ukraine; ceroform@gmail.com; 4 Institute of Physics, ELI Beamlines, Academy of Sciences of the Czech Republic, Na Slovance 2, CZ-18221 Prague, Czech Republic; borislav.angelov@eli-beams.eu; 5 Konstantinov Petersburg Nuclear Physics Institute NRC KI, Orlova Roscha, 188300 Gatchina, Leningrad district, Russia; kopitsa_gp@pnpi.nrcki.ru; 6 Grebenshchikov Institute of Silicate Chemistry of the Russian Academy of Sciences, Adm. Makarova emb. 2, 199155 St. Petersburg, Russia

**Keywords:** tungsten oxide, polyvinylpyrrolidone, ion exchange, photochromism, photocatalysis

## Abstract

Tungsten oxide-based bulk and nanocrystalline materials are widely used as photocatalytic and photo- and electrochromic materials, as well as materials for biomedical applications. In our work, we focused our attention on the effect of sodium cations on the structure and photochromic properties of the WO_3_@PVP aqueous sols. To establish the effect, the sols were synthesized by either simple pH adjusting of sodium or ammonium tungstates’ solutions, or using an ion exchange technique to remove the cations from the materials to the greatest possible extent. We showed that the presence of sodium cations in WO_3_@PVP favors the formation of reduced tungsten species (W^+5^) upon UV irradiation of the materials, strongly affecting their photochromic and photocatalytic properties. The pronounced photoreductive properties of WO_3_@PVP sols in photocatalytic reactions were demonstrated. Due to photoreductive properties, photochromic sols of tungsten oxide can act as effective photoprotectors in photooxidation processes. We believe that our work provides a considerable contribution to the elucidation of photochromic and redox phenomena in WO_3_-based materials.

## 1. Introduction

Tungsten oxide is a semiconductor material widely used in heterogeneous catalysis [1] and in photo- and electrochromic devices [2]. Tungsten oxide nanoparticles have also been considered a promising nanomaterial for biomedical applications: In recent years, WO_3_-based materials have been used in advanced medical and biological research as antibacterial coatings, biosensors, theranostic materials, and materials for proliferation control [3,4,5,6,7]. The majority of applications of these materials are due to the photochromic effect and the ability to participate in reversible redox reactions due to the W^+6^-W^+5^ transformations. Despite the fact that tungsten oxide-based bulk materials and thin films are well studied [8,9], the synthesis and photochromic properties of colloidal WO_3_ solutions in liquid media are still largely unexplored.

Several mechanisms have been proposed to explain the photochromism of WO_3_. One of the earliest theories of WO_3_ photochromism declared that the positively charged oxygen vacancies in the WO_3_ crystal structure are able to capture photogenerated electrons [10]. Certainly, the oxygen effect on the optical and photochromic properties of materials based on WO_3_ is a common phenomenon [11]. Another commonly accepted theory of photochromism is based on the assumption of W^+5^ stabilization due to the hydrogen tungsten bronzes’ (H_x_W^VI^_1−x_W^V^_x_O_3_) formation [12]. This theory has several experimental confirmations; for instance, the correlation was confirmed between bronzes’ formation and photochromic effect in the mesoporous WO_3_·0.33H_2_O nanorods [13]. Physically adsorbed water plays a key role in the mechanism of photochromism in anhydrous oxides [14,15,16,17]. Under UV irradiation, electrons and holes are generated in tungsten oxide samples. Photogenerated holes can react with adsorbed water on the surface of the WO_3_ crystallites with H^+^ formation. Then, hydrogen ions diffuse into the WO_3_ structure, which leads to W^+5^ stabilization and the corresponding color change. The by-products in this reaction are both oxygen and hydroxyl radicals. Thus, the photochromism in WO_3_ materials is typically caused by the changes in the structure and in the composition of tungsten oxide [18].

Existing studies pay special attention to the enhancement of the photochromic properties of bulk materials based on tungsten oxide, with typical sizes of nanoparticles in the range from tens to hundreds of nanometers. In turn, the study of the photochromic processes in X-ray amorphous materials based on tungsten oxide or organic W^+6^ complexes have received much less attention. The main mechanism for the reduction of W^+6^ to W^+5^ is the electron transfer from an organic stabilizer or a ligand to tungsten ion [19]. The reduction of W^+6^ in the course of the photochromic response is supposed to be mainly due to the transfer of an electron from an organic stabilizer [20]. The photoexcitation of WO_3_ leads to the transfer of electron density from the oxygen atom to the tungsten atom. The presence of nitrogen atoms in the stabilizer molecule results in an increase in photochromic properties due to the increased donor ability of nitrogen [21,22].

The most general approach to the description of the photochromic properties of tungsten oxide-based materials has been proposed recently [23,24]. Under UV irradiation, both protons and electrons are intercalated into the WO_3_ structure that results in W^+6^ reduction and crystal structure stabilization due to bronzes’ formation. Thus, when polyvinylpyrrolidone (PVP) is used as a stabilizer, the PVP molecules act as effective electron donors, whereas H_2_O molecules act as the source of protons. Since the generally accepted mechanism of WO_3_ photochromism involves the formation of tungsten bronzes, the photochromic properties of WO_3_-based materials should also be affected by the incorporation of the single charged cations (e.g., Na^+^) in their structure. A literature survey shows that such an effect has not been studied in detail. In this paper, in order to elucidate the role of sodium cations in the photochromism of WO_3_@PVP sols, they were synthesized by either pH adjusting of sodium or ammonium tungstates’ solutions, or using ion exchange technique to remove sodium or ammonium cations from the materials to the greatest possible extent. The structure and photochromic properties of the obtained materials were carefully studied. Special attention was also paid to the interplay of the recently reported UV-assisted organic dyes’ discoloration in the presence of WO_3_@PVP water dispersible nanoparticles [21] and the strongly reducing properties of W^+5^ species, which form in the photochromic WO_3_ nanoparticles upon UV-irradiation. Surprisingly, we found that WO_3_@PVP nanoparticles possess photoreductive and photoprotective properties.

## 2. Results

All WO_3_ sols were synthesized using two different techniques: By using ion exchange technique and by direct acidification of sodium or ammonium tungstates’ aqueous solutions. Hereafter, the sample names Na_1, Na_2, and Na_3 correspond to the sols synthesized using the ion exchange technique from sodium tungstate solution with various polyvinylpyrrolidone (PVP) content (molar ratio WO_3_: PVP = 1:1; 1:2; 1:4, respectively). The sample names NH_1, NH_2, and NH_3 correspond to the sols synthesized using the ion exchange technique from ammonium metatungstate solution with various PVP content (molar ratio WO_3_: PVP = 1:1; 1:2; 1:4, respectively). The samples obtained using direct acidification of sodium or ammonium tungstate solutions are labeled hereafter as M(N)x, were symbol M designates the samples synthesized from ammonium metatungstate and symbol N designates the samples synthesized from sodium tungstate, x indicate an approximate pH of a solution.

### 2.1. Small- and Wide-Angle X-ray Scattering

**Figure 1:**
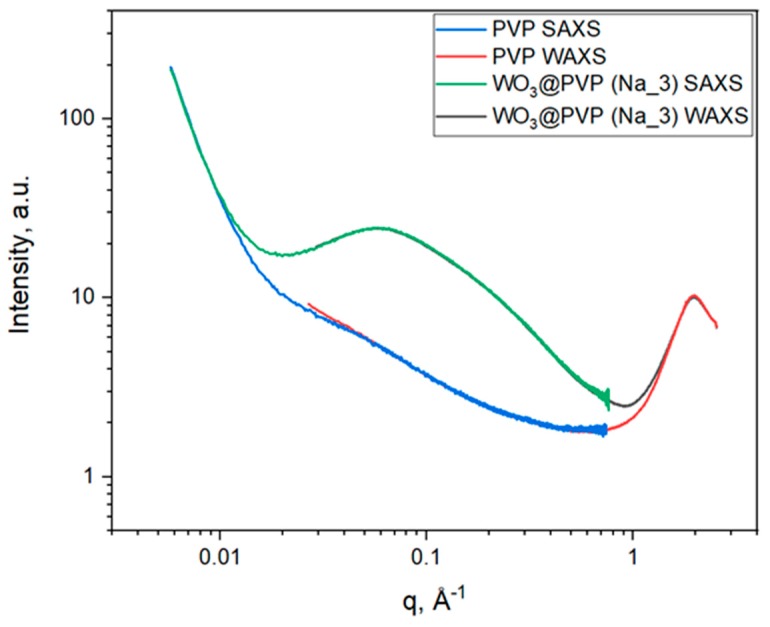
Results of small- and wide-angle X-ray scattering by PVP and WO_3_@PVP (Na_3) samples.

Analysis of the small-angle X-ray scattering (SAXS) and wide-angle X-ray scattering (WAXS) data (Figure 1) allows us to conclude that the structures of individual PVP or WO_3_@PVP composite differ significantly. Nevertheless, in the low and high q-ranges (*q* < 0.01 Å^−1^ and *q* > 1 Å^−1^ and), the scattering curves for the composite or the individual PVP coincide, which indicates that the scattering in these q-ranges was due to the PVP structure. This scattering pattern is typical for PVP; the peak at 2 Å^−1^ corresponds to closely packed side PVP chains, which have characteristic sizes of about 0.5 Å [25]. In the low q-range, up to 5 × 10^−3^ Å^−1^, there was no Guinier region, which indicates that the characteristic dimensions of PVP aggregates exceeded 200 nm.

The coincidence of the scattering patterns in the low and high q-ranges for PVP and WO_3_@PVP composite also indicates the colloidal stability of PVP-coated tungsten oxide particles and the absence of large aggregates. X-ray scattering from the WO_3_@PVP composite in the range from 0.01 Å^−1^ to 1 Å^−1^ with a maximum at 0.05 Å^−1^ corresponds to the particles of about 2 nm in size.

### 2.2. X-ray Diffraction

**Figure 2:**
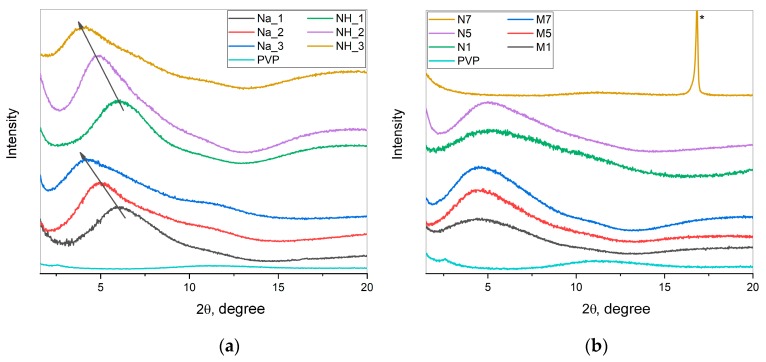
XRD patterns of dried WO_3_@PVP sols: (**a**) Samples obtained using the ion-exchange technique from sodium tungstate (Na_1, Na_2, and Na_3) and from ammonium metatungstate (NH_1, NH_2, and NH_3); (**b**) samples obtained by the pH adjusting from sodium tungstate (N1, N5, and N7) and from ammonium metatungstate (M1, M5, and M7). As a reference, the data for pure PVP (not modified with WO_3_ nanoparticles) are also given.

Figure 2 shows the X-ray diffraction (XRD) patterns of dried WO_3_@PVP samples along with the XRD pattern of the dried PVP solution. XRD patterns of dried WO_3_@PVP sols prepared by ion exchange demonstrated an intense scattering peak at small angles, which was not observed in the XRD pattern of individual PVP. This peak corresponded to the scattering from WO_3_ nanoparticles confined in the dried WO_3_@PVP composite. It was clearly seen that the position of the peak and, consequently, the size of the obtained particles were in a strict correlation with the concentration of PVP: The increase in the amount of PVP led to the formation of larger WO_3_ particles. For example, when a mass ratio of WO_3_:PVP was 1:1, the size of tungsten oxide particles was about 1.4 nm, whereas an increase in the WO_3_:PVP ratio to 1:4 resulted in 2 nm WO_3_ particles formation. At pH 7, the formation of a composite was not observed, which was confirmed by the absence of a peak at small angles. The diffraction pattern for the N7 sample coincided with the diffraction pattern of individual PVP. The diffraction maximum in the XRD pattern of the N7 sample at 16.8°2θ corresponded to the [111] peak of the Na_2_WO_4_ phase (card [12–772], PDF-2, ICDD database).

In the case of the samples obtained without the use of the ion exchange technique, the formation of the particles of approximately the same size (about 1.8 nm) was observed regardless of the pH value.

Thus, it was confirmed that all the dried samples except N7 contained WO_3_ nanoparticles.

### 2.3. Fourier-Transform Infrared Spectroscopy

**Figure 3:**
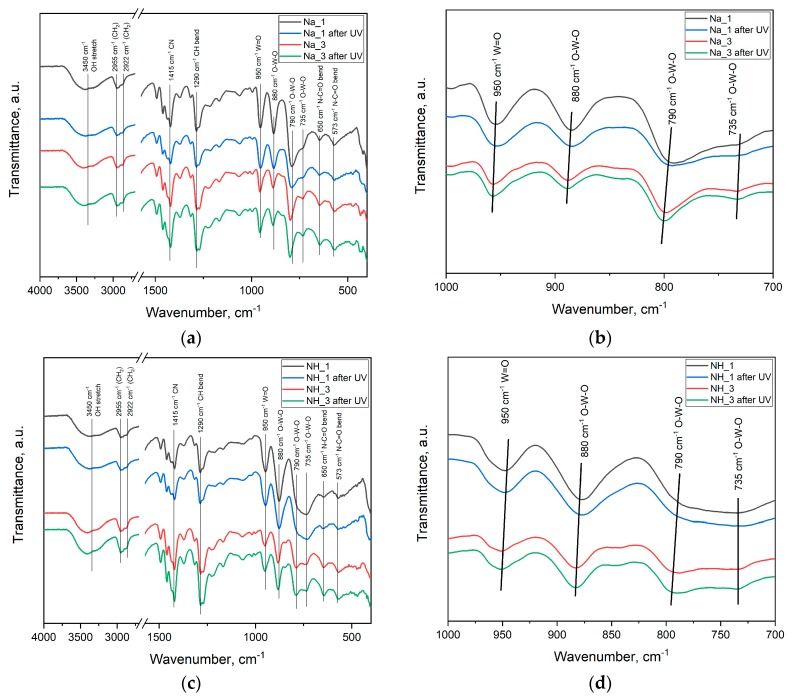
FTIR spectra of dried WO_3_ sols synthesized from sodium tungstate by the ion-exchange technique (Na_1 and Na_3): (**a**) Survey spectrum, (**b**) spectrum in the range of 1000–700·cm^−1^. FTIR spectra of dried WO_3_ sols synthesized from ammonium metatungstate by the ion-exchange technique (NH_1 and NH_3): (**c**) Survey spectrum, (**d**) spectrum in the range of 1000–700·cm^−1^.

Fourier-transform infrared (FTIR) spectra of dried WO_3_ sols are shown in Figure 3. The FTIR spectra of dried WO_3_ sols are identical to the spectrum of individual PVP presented elsewhere [26], excepting the ranges of 795–995 cm^−1^ and 420–435 cm^−1^. Absorbance in these ranges is typical for tungsten oxide [27,28,29]. FTIR spectra of dried WO_3_ sols irradiated with UV light (λ = 312 nm, exposure time of 10 min) were similar to the spectra of dried WO_3_ sols kept in the dark, while a slight difference in the splitting of the absorption band at 430 cm^−1^ was observed. Such a difference can be due to the distortions of [WO_6_] octahedra caused by the changes in the tungsten oxidation state. It should also be noted that the absorption band at 795 cm^−1^ for the sols synthesized from ammonium metatungstate was in all cases broader than the same band for the sols obtained from sodium tungstate. Apparently, this can be related to the influence of the cation: Ammonium had greater ionic radius than sodium for the same coordination numbers [30,31].

### 2.4. Transmission Electron Microscopy

**Figure 4:**
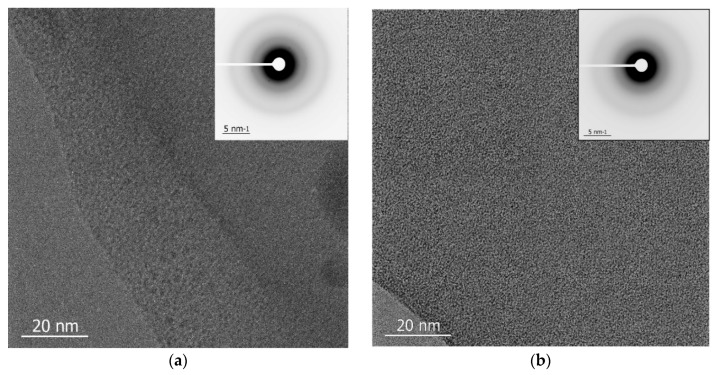
TEM images of: (**a**) NH_3 and (**b**) Na_3 samples. Electron diffraction patterns are shown in the insets.

Figure 4 shows transmission electron microscopy (TEM) images of WO_3_@PVP (Na_3 and NH_3) composites consisting of the particles with the specific sizes of several nanometers. The electron diffraction images do not contain any reflexes that prove that the samples are amorphous.

### 2.5. Photochromic Tests

**Figure 5:**
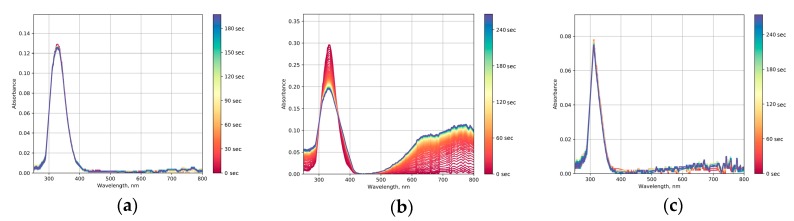
Optical absorption spectra of: (**a**) N1, (**b**) N5, and (**c**) N7 sols upon UV irradiation.

To confirm the tungsten oxide formation and to compare the photochromic properties of all the obtained samples, the evolution of optical absorption spectra upon UV irradiation was analyzed. Figure 5 shows the time dependences of the absorption spectra of WO_3_@PVP composites synthesized at different pH values. The optical absorption spectrum of the N7 sample was identical to the individual PVP spectrum, and the sample did not exhibit photochromic properties. This indicates that the formation of the WO_3_@PVP composite did not occur at pH 7, which was also confirmed by the results of the XRD analysis. In the case of N5 and N1 samples, the absorption edge differed from the PVP absorption edge and was shifted to the larger wavelengths range, which confirmed the formation of tungsten oxide in these syntheses. The N5 sample demonstrated the pronounced photochromic properties, while the N1 sample only demonstrated poor photochromism, probably due to poor redox activity of WO_3_ nanoparticles in an acidic media.

All the sols obtained from ammonium metatungstate did not exhibit the photochromic effect, and their absorption spectra were identical to those of the PVP solution. Thus, we can assume that under these conditions ammonium metatungstate was stable and it did not transform into tungsten oxide. Nevertheless, when these sols were dried, tungsten oxide nanoparticles were finally formed, as confirmed by the results of XRD analysis.

**Figure 6:**
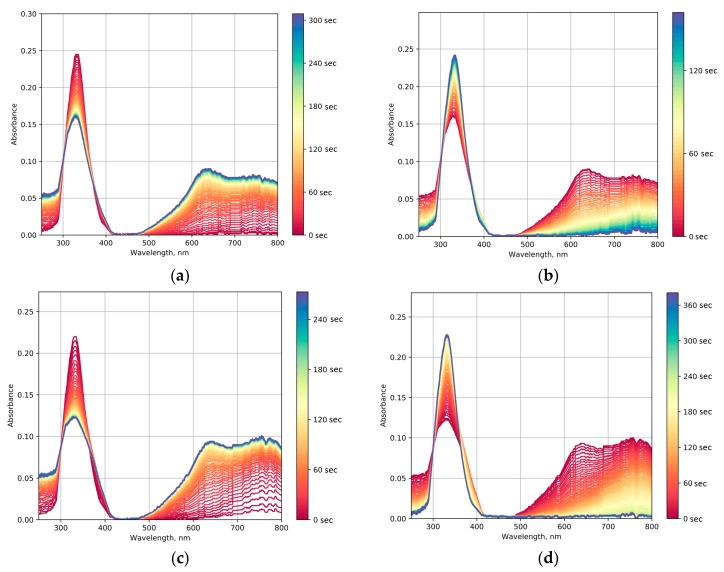
Time-resolved optical absorption spectra of the Na_2 sol: (**a**) Under UV irradiation, (**b**) after switching off the UV source. Time-resolved optical absorption spectra of the Na_3 sol: (**c**) Under UV irradiation, (**d**) after switching off the UV source.

**Figure 7:**
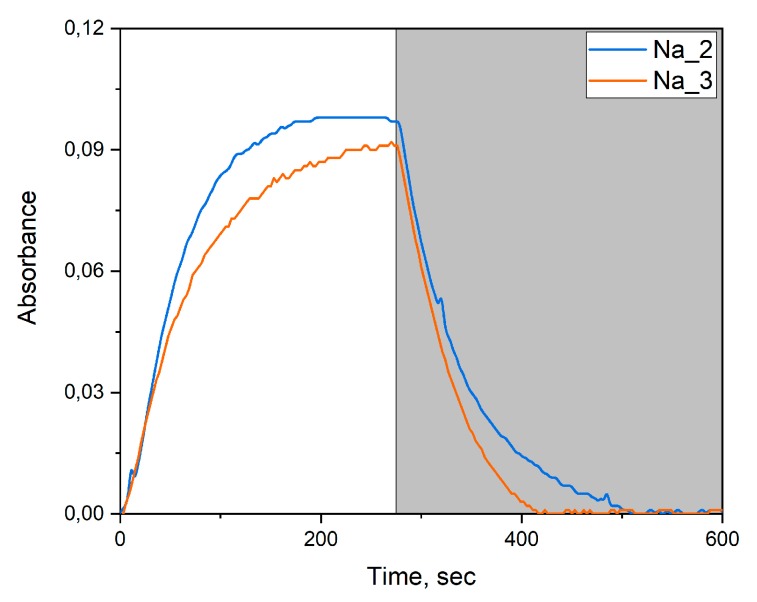
Dependence of Na_2 and Na_3 sols’ absorption intensity at 630-nm wavelength on irradiation time. The light area of the plot, UV light is switched on; the dark area of the plot, UV light is switched off.

In the syntheses with the ion-exchange resin and sodium tungstate as the precursor, WO_3_@PVP composites were formed, which was confirmed by the changes in optical absorption spectra and by the appearance of the photochromic effect. Our experimental data indicated that upon the increase in the PVP to WO_3_ ratio, the photochromic effect became less pronounced (Figure 6). This was manifested by a lower rate of coloration and faster discoloration after switching off UV irradiation (Figure 7). The differences in photochromic behavior of the samples most probably arose from the size effect as the increase in the amount of PVP added to the sol resulted in the larger WO_3_ particles.

**Figure 8:**
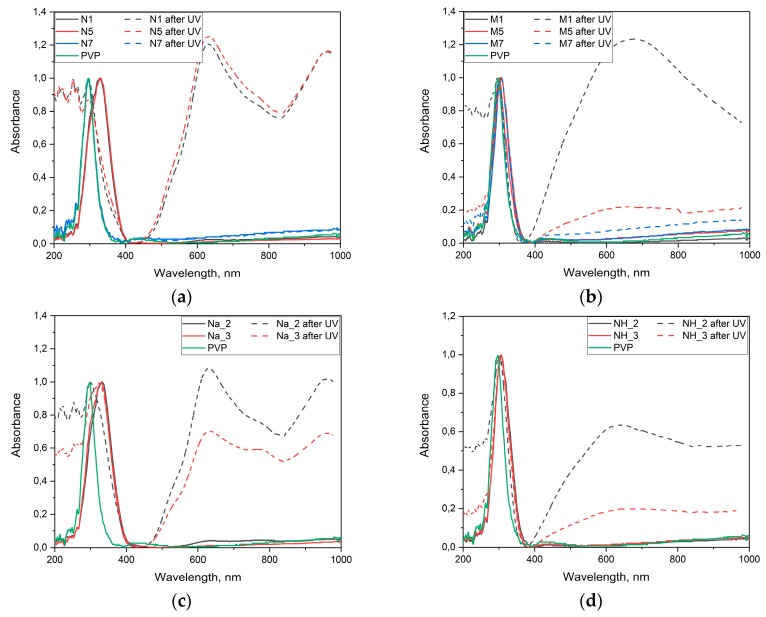
Optical absorption spectra of dried WO_3_@PVP samples before and after UV irradiation for 20 min: (**a**) Samples obtained at different pH values from sodium tungstate without ion-exchange treatment, (**b**) samples obtained at different pH values from ammonium metatungstate without ion-exchange treatment, (**c**) samples obtained from sodium tungstate using ion-exchange treatment, (**d**) samples obtained from ammonium metatungstate using ion-exchange treatment.

Figure 8 shows the absorption spectra of dried WO_3_@PVP samples before and after UV irradiation. Peaks at 630 and 750 nm were present in the optical absorption spectra of all the samples prepared from sodium tungstate. In turn, the spectra of the samples obtained from ammonium metatungstate differed significantly from those described above. Here, there were no narrow peaks in the spectra, and the wide absorption band was present in the range from 400 to 1000 nm. This indicates possible formation of ammonium tungsten bronzes with a different absorption spectra. The appearance of dried sols synthesized from sodium tungstate and ammonium metatungstate before and after UV irradiation is shown in Figure S2.

The photochromic properties of the samples correlated with the results of XRD analysis. For all the samples having a small-angle diffraction maximum in the XRD pattern, the photochromic effect was observed.

**Figure 9:**
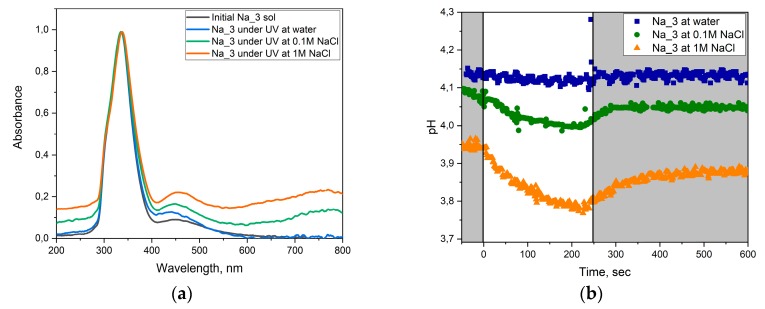
(**a**) Optical absorption spectra of the Na_3 sol in deionized water and in NaCl solutions. (**b**) Time dependences of Na_3 sol pH values. The light area of the plot, UV light is switched on; the dark area of the plot, UV light is switched off.

In order to determine the mechanism of photochromism we continuously measured the pH of the solution during the photochromic cycling of the Na_3 sample. These experiments were carried out both in deionized water and in 0.1 M and 1 M NaCl solutions (Figure 9).

According to the data obtained (Figure 9b), UV irradiation of the Na_3 sol was accompanied with a decrease in the sample pH, while after turning off the UV irradiation the pH value was almost completely recovered. Upon the increase in NaCl concentration, this effect also increased. At the same time, there were nearly no changes in the pH value registered in the experiments performed in deionized water.

The mechanism of photochromism described in the current literature is presumably based on the formation of hydrated tungsten oxide in the reaction with H^+^:

(1) $$xH^{+}+xe^{-}+{\mathrm{WO}}_{3}\to H_{x}W_{1-x}^{+6}W_{x}^{+5}O_{3}$$

(2) $$x{\mathrm{Na}}^{+}+xe^{-}+{\mathrm{WO}}_{3}\to {\mathrm{Na}}_{x}W_{1-x}^{+6}W_{x}^{+5}O_{3}.$$

As follows from Equation (1), in the absence of alkali metal cations, the formation of hydrated tungsten oxide should increase the solution pH, which was not observed in our experiments. However, according to Equation (2), a decrease in the pH values can take place due to the formation of sodium tungsten bronzes. High stability of tungsten bronzes leads to a relatively larger fraction of the tungsten reduced form (W^+5^), as can be seen from the optical absorption spectra (absorption at 600–800 nm rises with an increase in the concentration of sodium cations in the solution).

### 2.6. Photocatalytic Dye Discoloration

**Figure 10:**
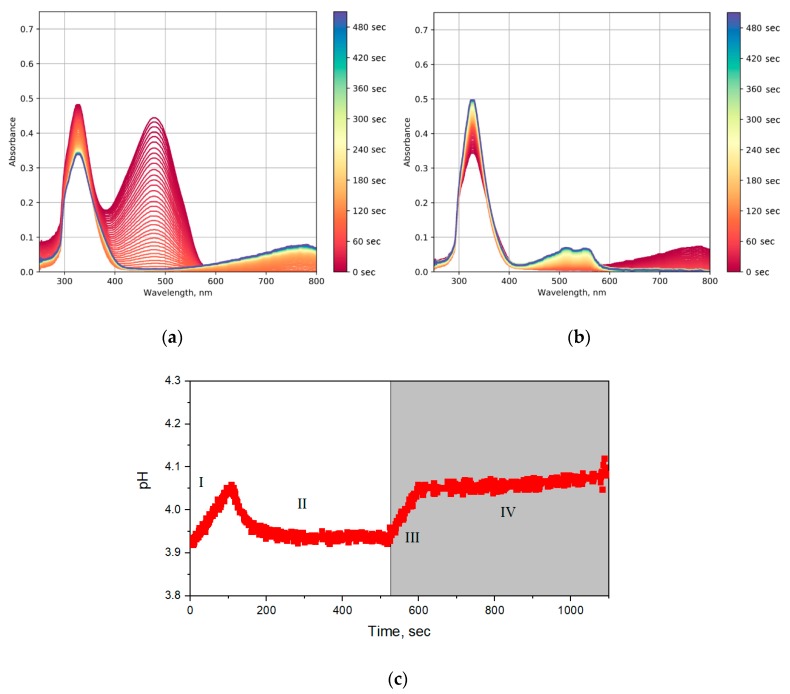
Optical absorption spectra of a mixture of WO_3_ sol and methyl orange: (**a**) Under UV irradiation, and (**b**) after turning off UV irradiation. (**c**) Time dependence of pH during the photocatalytic reaction. The light area of the plot, UV light is switched on; the dark area of the plot, UV light is switched off.

In order to study the photocatalytic properties of the obtained tungsten oxide sols, the rate of methyl orange solution discoloration was determined. The corresponding time-resolved optical absorption spectra are shown in Figure 10. At the first stage, up to ~120 s, the color of the solution was faded, which was accompanied by an increase in pH value (I in Figure 10c). After that, photochromism was observed, accompanied by an increase in the intensity of absorption at wavelengths of 600–800 nm (Figure 10a) and a decrease in pH (II in Figure 10c). After switching off the UV source, the sol bleached in ~100 s (Figure 10b and III in Figure 10c). After that, two absorption peaks occurred at 510 and 560 nm, which may correspond to the acidic form of methyl orange (Figure 10b and IV in Figure 10c).

Photocatalytic decomposition of methyl orange in the presence of oxide photocatalysts is known to proceed through an oxidative mechanism involving the hydroxyl radicals and photogenerated holes. Under anaerobic conditions, the reductive discoloration of methyl orange (MO) proceeds. This reaction is accompanied by methyl orange transformation into a hydrazine derivative [32]:

(3) $${\left({\mathrm{CH}}_{3}\right)}_{2}{\mathrm{NC}}_{6}H_{4}N\;=\;{\mathrm{NC}}_{6}H_{4}{\mathrm{SO}}_{3}^{-}+2H^{+}+2e^{-}\to {\left({\mathrm{CH}}_{3}\right)}_{2}{\mathrm{NC}}_{6}H_{4}{\mathrm{NHNHC}}_{6}H_{4}{\mathrm{SO}}_{3}^{-}$$

The obtained hydrazine derivative is unstable in the absence of UV radiation, and, as a consequence, in the absence of the reducing agent (W^+5^). After turning the UV irradiation off, hydrazine derivative disproportionate to form methyl orange, its acidic form can be traced in the optical absorption spectra [33]:

(4) $$\begin{array}{c} 2{\left({\mathrm{CH}}_{3}\right)}_{2}{\mathrm{NC}}_{6}H_{4}{\mathrm{NHNHC}}_{6}H_{4}{\mathrm{SO}}_{3}^{-}\;\to \;{\left({\mathrm{CH}}_{3}\right)}_{2}{\mathrm{NC}}_{6}H_{4}N\\ \;={\mathrm{NC}}_{6}H_{4}{\mathrm{SO}}_{3}^{-}+{\left({\mathrm{CH}}_{3}\right)}_{2}{\mathrm{NC}}_{6}H_{4}{\mathrm{NH}}_{2}+H_{2}{\mathrm{NC}}_{6}H_{4}{\mathrm{SO}}_{3}^{-} \end{array}$$

**Figure 11:**
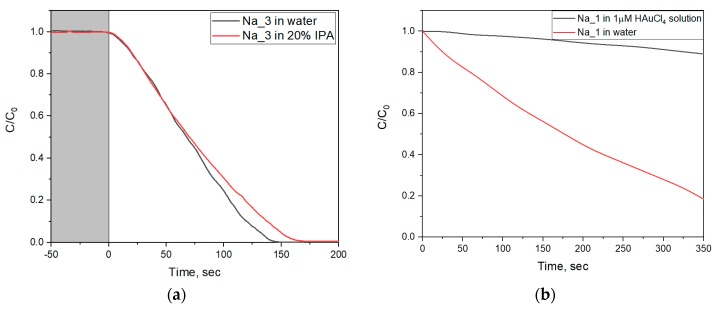
(**a**) Time dependence of methyl orange concentration during WO_3_@PVP-assisted photocatalytic decomposition in water and in IPA solution. The light area of the plot, UV light is switched on; the dark area of the plot, UV light is switched off. (**b**) Time dependences of methylene blue concentration upon UV irradiation in the presence of WO_3_@PVP (sample Na_1) or in the presence of WO_3_@PVP (sample Na_1) and 1 μM HAuCl_4_.

The reductive photodegradation of methyl orange is also confirmed by the absence of the influence of isopropyl alcohol (IPA) on the photodegradation rate. IPA is one of the most widely used inhibitors of photocatalytic processes, due to its reaction with hydroxyl radicals [34]. Figure 11a shows time-resolved methyl orange absorption spectra during methyl orange photodecomposition by WO_3_@PVP sols both in deionized water and in a 20% IPA solution. For the photocatalytic decomposition of MO in water, the discoloration rate was 0.46 g^−1^·s^−1^, whereas in the 20% IPA solution, the rate was 0.42 g^−1^·s^−1^, which corresponded to a decrease in the photodegradation constant by 9%. A minor difference in the reaction rates shows that hydroxyl radicals did not take part in the discoloration process. Thus, the experiment with IPA showed that oxidizing agents did not contribute to the photocatalytic degradation of the organic dye.

The reducing mechanism of photocatalytic activity of WO_3_@PVP nanoparticles was additionally confirmed by the introduction of HAuCl_4_ in the reaction mixture. Here, the reduction of HAuCl_4_ and the formation of gold nanoparticles was a competitive process and contributed to the organic dye discoloration rate [34]. Since the gold nanoparticles cause plasmon resonance effect, their absorption may interfere with the absorption of the methyl orange. Therefore, in the corresponding photocatalytic experiments, we used a methylene blue dye (MB) as a model organic dye.

Time dependences of MB concentration in the presence of individual WO_3_@PVP or a mixture of WO_3_@PVP and HAuCl_4_ are presented in Figure 11b. The data show that the introduction of HAuCl_4_ (1 μM) inhibited the discoloration process caused by the WO_3_@PVP sol. The constant of the photodegradation process in the presence of WO_3_@PVP and HAuCl_4_ was only 8% of the rate of MB discoloration in the presence of bare WO_3_@PVP nanoparticles. This experiment also confirmed the proposed reducing mechanism of organic dyes’ discoloration in the presence of WO_3_@PVP nanoparticles.

**Figure 12:**
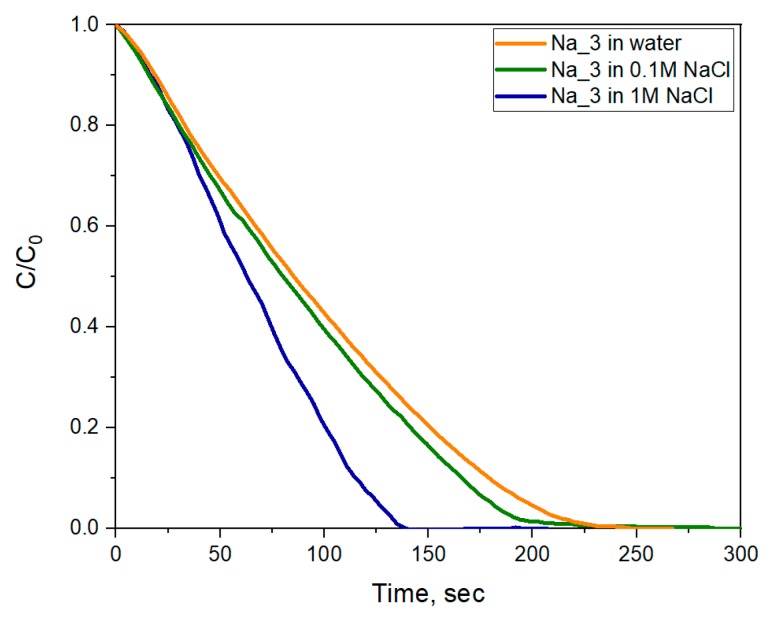
Comparison of the time dependences of methyl orange photodegradation by the Na_3 WO_3_@PVP sol at different NaCl concentrations.

Analysis of the time dependence of the MO concentration showed an increase in the rate of discoloration with an increase in NaCl concentration (Figure 12). In a 0.1 M NaCl solution, the MO discoloration rate for the Na_3 sample increased from 0.46 s^−1^·g^−1^ to 0.48 s^−1^·g^−1^, and in a 1 M NaCl solution it increased to 0.66 s^−1^·g^−1^. As mentioned above, the increase in sodium concentration led to the formation of much more stable sodium tungsten bronzes and, subsequently, to the enhancement of the photochromic properties. The higher fraction of W^+5^ associated with the increased concentration of sodium cations resulted in an increase in the rate of photoreductive discoloration of methyl orange.

**Figure 13:**
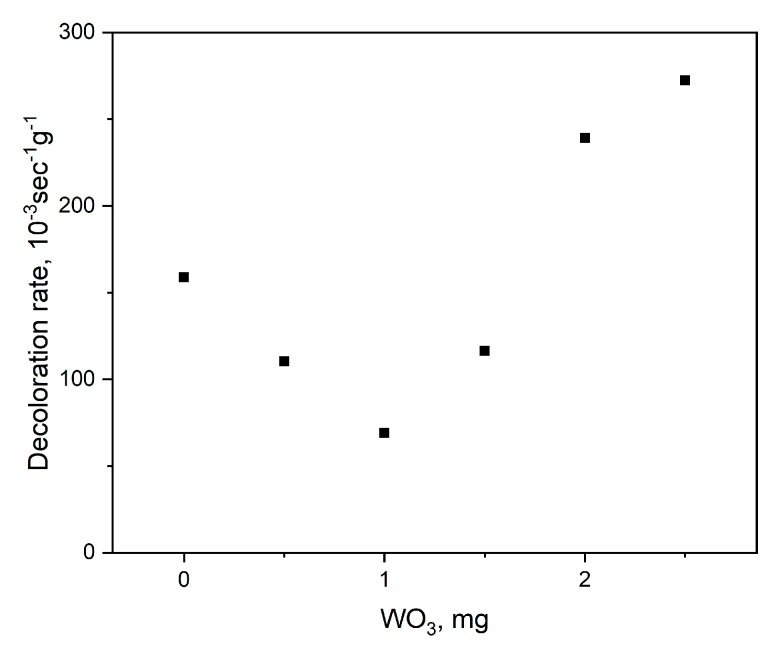
Photoprotective properties of the Na_3 WO3@PVP sol in the methyl orange photooxidation reaction induced by a TiO_2_ photocatalyst.

Due to their high photoreduction ability, obtained WO_3_@PVP samples can be used as active photooxidation protectors. Thus, methyl orange discoloration rate by the Aeroxide P25 commercial photocatalyst (TiO_2_, 25% rutile, 75% anatase) depended on the concentration of the Na_3 sol. As can be seen from Figure 13, the increase in WO_3_ concentration led to the decrease in the methyl orange photocatalytic decomposition rate, which was associated with the photoreduction of hydroxyl radicals formed upon UV irradiation of TiO_2_. Here, the main mechanism of photodegradation was still the oxidative decomposition of methyl orange. The further increase in WO_3_ content resulted in the increase in the discoloration rate, due to the predominance of photoreductive processes on WO_3_ particles.

## 3. Materials and Methods

### 3.1. Materials

Sodium tungstate dihydrate (Na_2_WO_4_ ≥ 99%, Sigma-Aldrich, Darmstadt, Germany), ammonium metatungstate hydrate ((NH_4_)_6_H_2_W_12_O_40_·xH_2_O ≥ 99%, Sigma-Aldrich), polyvinylpyrrolidone (PVP K-30, average mol. wt. 40,000), HCl, NaOH, NaCl (99%, Sigma-Aldrich), methyl orange (MO, C14H14N3NaO3S, Arcos), cation exchange resin (Amberlite IR120, Supelco Inc., Bellefonte, PA, USA), and TiO_2_ (Aeroxide^®^ P25, ≥99.5%, Evonik, Essen, Germany) were used as received.

### 3.2. Preparation of Sols

Hydrated tungsten oxide nanoparticles stabilized with PVP were synthesized by a wet chemistry technique. The first series of samples was obtained by the method reported earlier [18]. Briefly, tungstic acid was synthesized by an ion-exchange technique using sodium tungstate (Na_2_WO_4_) solution and strongly acidic cation exchange resin (Amberlite IR120). Ion-exchange resin was swelled in water and loaded into a 200 mL glass column. Then, 100 mL of 0.05 M sodium tungstate solution was passed through the column dropwise. After that 1, 2, or 4 g of PVP was added to the obtained eluent to prepare Na_1, Na_2, and Na_3 samples, correspondently. The solution was transferred to the flask and stirred for 4 h under 80 °C. The thus formed sol turned out to be stable, while a precipitate was formed after several hours when the ion-exchange procedure was performed without the addition of PVP.

Using ammonium metatungstate instead of sodium tungstate, samples NH_1, NH_2, and NH_3 were obtained.

The second series of samples was prepared without the use of the ion-exchange resin. To 100 mL of 0.05 M sodium tungstate solution or 0.05 M ammonium metatungstate solution, 4 g of PVP were added, then the pH value was adjusted to 1, 5, or 7, using 0.1 M HCl or NaOH, which were added dropwise. Thus prepared solutions were stirred for 4 h at 80 °C to obtain the sols. The samples were marked M(N)x, where symbol M designated the samples synthesized from ammonium metatungstate, symbol N designated the samples synthesized from sodium tungstate, and x indicated an approximate pH value of the solution.

### 3.3. Characterization

#### 3.3.1. XRD

X-ray diffraction (XRD) patterns were recorded using a Rigaku D/MAX 2500 diffractometer (θ/2θ Bragg-Brentano reflection geometry) with a scintillation counter. All the measurements were performed with CuKα1,2 radiation generated on a rotating Cu anode (50 kV, 250 mA) and monochromatized by a curved graphite [002] monochromator placed at the reflected beam. The XRD patterns were collected in the 1.5–20° 2θ range with a 0.02° step and at least 5 s/step. To reduce the undesirable background intensity, all dried samples were investigated on monocrystalline [510] Si holders.

#### 3.3.2. SAXS

Small-angle and wide-angle X-ray scattering experiments were performed at the SWING beamline of synchrotron SOLEIL (Saint Aubin, France). To measure scattering from 5 × 10^−3^ to 3 Å^−1^, the sample-to-detector distances were 0.5–2 m. The patterns were recorded with a two-dimensional EigerX 4-M detector (Dectris, Baden, Switzerland). The synchrotron radiation wavelength was λ = 0.775 Å. The investigated samples were placed in capillaries with a diameter of 1.5 mm and were sealed by paraffin wax. Scattering patterns of an empty capillary and a capillary filled with MilliQ water were recorded for intensity background subtraction. Data processing of the recorded 2D images was performed by FOXTROT software (version 3.2.7, SOLEIL, France) [35].

#### 3.3.3. TEM

TEM images were acquired using a charge-coupled device (CCD) camera, Ultra Scan 4000 (Gatan, Gatan, Pleasanton, CA, USA), installed in a transmission electron microscope, Zeiss Libra 200 FE operated at 200 kV. The samples were applied to a copper grid covered with lacey carbon.

#### 3.3.4. FTIR

The FTIR spectra of the samples were recorded on a Bruker ALPHA spectrometer, in a range of 400–4000 cm^−1^, in attenuated total reflectance mode. To avoid solvent effect, WO_3_ sols were dried at 50 °C for 3 h.

#### 3.3.5. Photochromic and Photocatalytic Measurements

Photochromic and photocatalytic experiments were carried out in the flow-type measuring system. WO_3_@PVP sols were irradiated using a high-pressure mercury lamp with a power of 5.5 W. The complete scheme of the setup is described elsewhere [36]. The suspension absorption spectra were measured using an HRX-2000 xenon lamp, an Ocean Optics QE65000 spectrophotometer, and Ocean Optics fiber optics. The pH was controlled using an ESC-10314/7 combined electrode. The scheme of the experimental setup is presented in Figure S1.

In the typical experiment, 1 mL of the WO_3_ sol was added to 25 mL of deionized water, and after that the obtained solution was transferred in the measuring setup.

The study of organic dyes’ discoloration in the presence of WO_3_@PVP sols and in the WO_3_@PVP-TiO_2_ system was carried out under similar conditions. Methyl orange solution was used as a model dye. NaCl solutions of different concentrations were used to determine the effect of sodium concentration on the photodegradation rate. Photocatalytic decomposition of methyl orange in the presence of WO_3_@PVP-TiO_2_ was analyzed in a phosphate buffer solution at a fixed pH value of 6.86. In the photocatalytic experiments, the discoloration rate of MO was determined as the first-order reaction:

(5) $$\ln\left(\frac{C}{C_{0}}\right)\;=\;-K_{1}t$$

The discoloration rate (*K*_1_) was evaluated as the slope of the time dependence of MO relative concentration in semilogarithmic scale [37,38,39,40]. In all the cases, an approximation by a first-order reaction Equation (5) resulted in an *R*^2^ factor higher than 0.97.

Optical absorption spectra were recorded at intervals of 3 s. Absorption spectra were processed using Python software, using scipy and matplotlib libraries [41,42].

## 4. Conclusions

In our work, we focused our attention on the cation effect on the structure and photochromic properties of the WO_3_@PVP aqueous sols. WO_3_@PVP (polyvinylpyrrolidone) photochromic aqueous sols can only be prepared by slight acidifying of sodium tungstate solutions, while acidifying of ammonium metatungstate solutions results in nonphotochromic sols. The most probable reason for this effect is the high stability of polytungstate anions. In turn, drying of the sols (both synthesized from sodium tungstate and ammonium metatungstate) resulted in photochromic films due to the formation of tungsten oxide nanoparticles. An increase in PVP content resulted in a decrease in photochromic properties of the materials due to the formation of larger WO_3_ nanoparticles.

The presence of sodium cations in WO_3_@PVP materials favored formation of reduced tungsten species (W^+5^) upon UV irradiation, strongly affecting their photochromic and photocatalytic properties. This effect was probably due to the formation of stable Na-tungsten bronzes. When the WO_3_@PVP sols were synthesized using an ion-exchange method, which allowed minimizing sodium content in the sols, they demonstrated far less photochromic activity.

Finally, we proposed an experimental approach, which allowed us to establish photoprotective properties of WO_3_@PVP sols in photocatalytic reactions. Due to their photoreductive properties, photochromic sols of tungsten oxide can act as effective photoprotectors in photooxidation processes.

## Supplementary Materials

The following are available online at https://www.mdpi.com/1420-3049/25/1/154/s1, Figure S1: A scheme of the set-up used for photochromic measurements, Figure S2: Appearance of dried sols before and after UV irradiation.
