# Supplementary material for: Photochromic and Photocatalytic Properties of Ultra-Small PVP-Stabilized WO3 Nanoparticles
Source: Molecules. 2019 Dec 30;25(1):154. doi: 10.3390/molecules25010154 (PMC6982781; doi:10.3390/molecules25010154)
Supplement: Supplementary file 1 [file molecules-25-00154-s001.pdf]

Supplementary Materials

Photochromic and Photocatalytic Properties of Ultra-Small PVP-Stabilized WO_3 Nanoparticles

Daniil A. Kozlov [1,2], Alexander B. Shcherbakov [3], Taisiya O. Kozlova [1,2], Borislav Angelov [4], Gennady P. Kopitsa [5,6], Alexey V. Garshev [1], Alexander E. Baranchikov [1,2], Olga S. Ivanova [2] and Vladimir K. Ivanov [2,*]

1 Lomonosov Moscow State University, Leninskiye Hills 1, Moscow 119234, Russia; kozlov@inorg.chem.msu.ru (D.A.K.); taisia.shekunova@yandex.ru (T.O.K.); garshev@inorg.chem.msu.ru (A.V.G.); a.baranchikov@yandex.ru (A.E.B.)

2 Kurnakov Institute of General and Inorganic Chemistry of the Russian Academy of Sciences, 31 Leninsky av., Moscow 119991, Russia; runetta05@mail.ru

3 Zabolotny Institute of Microbiology and Virology, National Academy of Sciences of Ukraine, Kyiv, D0368 Ukraine; ceroform@gmail.com

4 Institute of Physics, ELI Beamlines, Academy of Sciences of the Czech Republic, Na Slovance 2, CZ-18221 Prague, Czech Republic; borislav.angelov@eli-beams.eu

5 Konstantinov Petersburg Nuclear Physics Institute NRC KI, Orlova Roscha, Gatchina 188300, Leningrad district, Russia; kopitsa_gp@pnpi.nrcki.ru

6 Grebenshchikov Institute of Silicate Chemistry of the Russian Academy of Sciences, Adm. Makarova emb., 2, St. Petersburg 199155, Russiae

* Correspondence: van@igic.ras.ru; Tel.: +7-495-952-0224

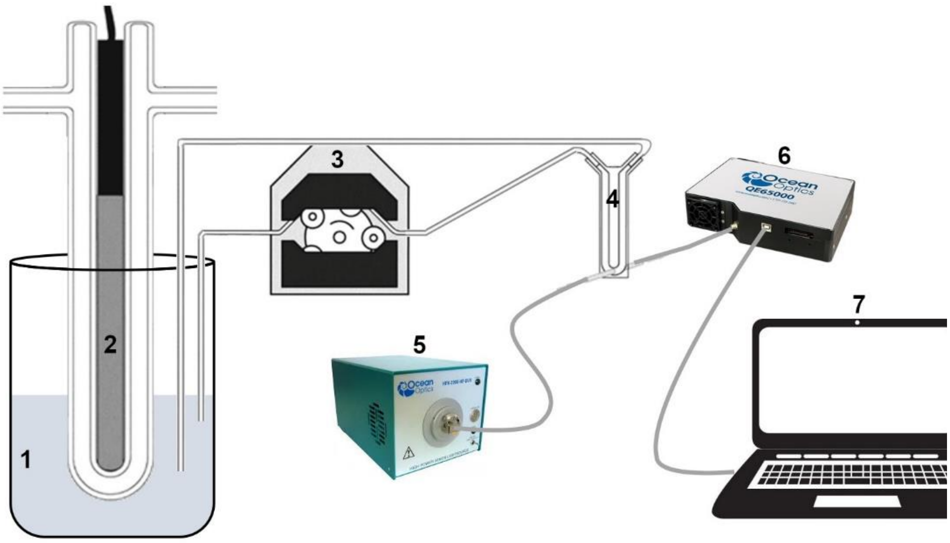

Figure 1. A scheme of the set-up used for photochromic measurements. 1 – WO3@PVP sol; 2 – high pressure UV mercury lamp (5.5 W); 3 – peristaltic pump; 4 – U-shaped measurement cuvette; 5 - HRX-2000 xenon lamp; 6 - Ocean Optics QE65000 spectrophotometer; 7 – laptop.

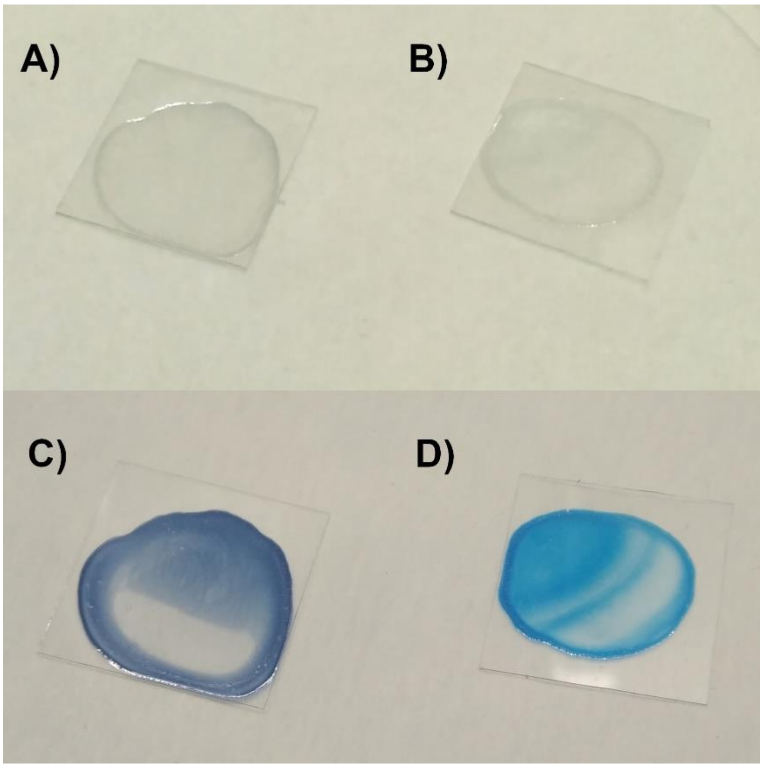

Figure S2. Appearance of dried (A,C) NH_2, and (B,D) Na_2 sols (A,B) before and (B,D) after UV irradiation.
